# Cryptic diversity found in Didymellaceae from Australian native legumes

**DOI:** 10.3897/mycokeys.78.60063

**Published:** 2021-02-08

**Authors:** Elizabeth C. Keirnan, Yu Pei Tan, Matthew H. Laurence, Allison A. Mertin, Edward C.Y. Liew, Brett A. Summerell, Roger G. Shivas

**Affiliations:** 1 School of Agriculture, Food and Wine, Waite Research Institute, The University of Adelaide, SA 5005, Australia The University of Adelaide Adelaide Australia; 2 Department of Agriculture and Fisheries, Ecosciences Precinct, Dutton Park, QLD 4102, Australia Royal Botanic Gardens and Domain Trust Sydney Australia; 3 Australian Institute of Botanical Science, Royal Botanic Gardens and Domain Trust, Mrs Macquaries Rd, Sydney, NSW 2000, Australia Department of Agriculture and Fisheries, Ecosciences Precinct Dutton Park Australia; 4 Centre for Crop Health, University of Southern Queensland, Toowoomba, QLD 4350, Australia University of Southern Queensland Toowoomba Australia

**Keywords:** Alternative host, multilocus phylogeny, pathogen reservoir

## Abstract

*Ascochyta koolunga* (Didymellaceae, Pleosporales) was first described in 2009 (as *Phoma koolunga*) and identified as the causal agent of Ascochyta blight of *Pisum sativum* (field pea) in South Australia. Since then *A. koolunga* has not been reported anywhere else in the world, and its origins and occurrence on other legume (Fabaceae) species remains unknown. Blight and leaf spot diseases of Australian native, pasture and naturalised legumes were studied to investigate a possible native origin of *A. koolunga*.

*Ascochyta koolunga* was not detected on native, naturalised or pasture legumes that had leaf spot symptoms, in any of the studied regions in southern Australia, and only one isolate was recovered from *P. sativum*. However, we isolated five novel species in the Didymellaceae from leaf spots of Australian native legumes from commercial field pea regions throughout southern Australia. The novel species were classified on the basis of morphology and phylogenetic analyses of the internal transcribed spacer region and part of the RNA polymerase II subunit B gene region. Three of these species, *Nothophoma garlbiwalawarda***sp. nov.**, *Nothophoma naiawu***sp. nov.** and *Nothophoma ngayawang***sp. nov.**, were isolated from *Senna artemisioides*. The other species described here are *Epicoccum djirangnandiri***sp. nov.** from *Swainsona galegifolia* and *Neodidymelliopsis tinkyukuku***sp. nov.** from *Hardenbergia violacea*. In addition, we report three new host-pathogen associations in Australia, namely *Didymella pinodes* on *S. artemisioides* and *Vicia cracca*, and *D. lethalis* on *Lathyrus tingitanus*. This is also the first report of *Didymella prosopidis* in Australia.

## Introduction

The Didymellaceae was established to accommodate *Ascochyta*, *Didymella*, and other allied *Phoma*-like genera (de Gruyter et al. 2009). To date, more than 5,400 species from 31 genera have been recorded, including recently established genera such as *Dimorphoma* and *Macroascochyta* (Hou et al. 2020). Species of Didymellaceae are cosmopolitan and occupy a broad range of environments. Many species are plant pathogens that cause leaf and stem lesions, often with a broad host range (Aveskamp et al. 2009; Aveskamp et al. 2010; Chen et al. 2015b). Multilocus phylogenetics and a polyphasic approach to classify species have helped to revise taxa and refine systematic relationships in the Didymellaceae (Aveskamp et al. 2009, de Gruyter et al. 2009; Aveskamp et al. 2010; Chen et al. 2015a, de Gruyter 2012; Hou et al. 2020).

In Australia, reports of taxa in the Didymellaceae mostly refer to plant pathogenic species, particularly on crop and pasture legumes (Fabaceae). In Australia, the disease Ascochyta blight of *Pisum sativum* (field pea) is typically caused by three fungal species, *Ascochyta koolunga*, *Didymella pinodella*, and *D. pinodes*. A fourth species, *Ascochyta pisi*, is very rarely isolated. One species in particular, *A. koolunga*, is an important part of the Ascochyta blight disease complex of field pea in South Australia (Davidson et al. 2009a). First described in 2009, *A. koolunga* (syn. *Phoma koolunga*) had spread across southern Australia and had been detected in Victoria and Western Australia by 2015 (Davidson et al. 2011; Tran et al. 2015a).

Molecular techniques are now routinely used to understand the genetic diversity and population structure of Didymellaceae (Aveskamp et al. 2010; Salam et al. 2011, de Gruyter 2012; Chen et al. 2015a, Hou et al. 2020). To date, there has not been a systematic inventory of leaf spot pathogens associated with Australian native legume species despite international reports from a diversity of countries on Ascochyta blight since 2009 (Le May et al. 2009; Mathew et al. 2010; Panicker and Ramraj 2010; Skoglund et al. 2011; Soylu and Dervis 2011; Gaurilcikiene and Viciene 2013; Liu et al. 2013; Ahmed et al. 2015; Liu et al. 2016). *Ascochyta koolunga* is only known to occur in Australia, which suggests an Australasian origin, with perhaps an association with native legume species. The aim of this study was to determine the species of Didymellaceae associated with leaf spot diseases, and to investigate possible native sources of *A. koolunga*. To this end we collected legume specimens from both cultivated and neighbouring natural ecosystems. In particular, we collected specimens from Australian native, pasture and naturalised legumes in the field pea growing regions of eastern and southern Australia.

## Materials and methods

### Sample collection and culturing

Samples of leaf tissue displaying leaf spot disease symptoms on legumes were obtained from 22 field pea trial sites, from the immediate surrounds of experimental and commercial crops and roadsides around crops in field pea growing regions of southern Australia. In total, 124 samples (stems with multiple leaves and more rarely seed pods and flowers) were collected during four separate 4–5 day (d) periods in August, September and October 2017. In addition to trial sites, local agronomists were contacted to obtain approval to allow access to growers’ properties in Eyre Peninsula (South Australia) and Horsham (Victoria).

The national parks, or conservation areas, nearest to the field pea sampling sites were identified prior to field trips and permits were obtained to enable collections of samples from native plants that exhibited leaf disease symptoms within these neighbouring natural ecosystems. Leaf disease samples were also collected from two botanic gardens, Adelaide Botanic Garden, Adelaide, South Australia and the Australian Botanic Garden, Mount Annan, New South Wales. Plants with leaf spots were photographed in the field with a Samsung galaxy S5 or S8 mobile phone camera and the GPS locations recorded. Representative leaf samples were placed in plastic bags, labelled and stored at 4 °C.

Within 5 d of collection, leaf specimens were surface disinfected by spraying with 70% v/v ethanol and blotted dry with fresh, non-sterilised tissue paper. Excised leaf pieces were placed on plates of potato dextrose agar (PDA) (Oxoid) acidified by supplementation with 1 ml of 85% v/v lactic acid per litre (APDA) to minimise bacterial contamination. Incubation was under a 12 hour (h) black and fluorescent light /12 h dark cycle at 22 °C for 7–10 d, when fungal colonies were examined microscopically for pycnidia and conidia. Representative isolates were subcultured onto PDA using hyphal tips and deposited in the culture collection of the Queensland Plant Pathology Herbarium (BRIP).

### DNA extraction, PCR and sequencing

Genomic DNA was extracted from 7 d old mycelium grown on PDA from the subculture isolates using the FastDNA Kit (Q-biogene Inc. Irvine, California, USA) according to the manufacturer’s instructions. A section of DNA from the internal transcribed spacer (ITS) region was amplified with the primers ITS1 and ITS4 (White et al. 1990), and the partial region of the RNA polymerase II subunit B (*rpb*2) gene was amplified with the primers RPB2-5F2 (Sung et al. 2007) and RPB2-7cR (Liu et al. 1999). The PCR conditions were as described by White et al. (1990) for ITS and O’Donnell et al. (2007) for *rpb*2. All PCRs were undertaken in 25 μl reaction volumes containing the final concentrations; 1 unit of PCR 5X buffer (Promega Corporation, Madison, Wisconsin, USA), 1.6 mM of 25 mM MgCl_2_ (Sigma-Aldrich Corporation, Louis, Missouri, USA), 0.025 U/μl of GoTaq™ (Promega), 0.6 mM of primer 1 and primer 2 and 1.6 mM of each dNTP (Promega). The PCR amplicons were purified using ExoSAP-IT (USB Corporation) following the manufacturer’s instructions. The purified amplicons were sent to the Ramaciotti Centre for Gene Function Analysis (University of New South Wales, Kensington, NSW), where DNA sequences were determined using an ABI PRISM 3700 DNA Analyser (Applied Biosystems Inc).

### Phylogenetic analysis

Forward and reverse sequences were assembled using Geneious v. 11.1.5 (Biomatters Ltd) and deposited in GenBank (Table 1, in bold). The sequences were aligned with selected reference sequences of Didymellaceae (Table 1) using the multiple alignment MAFFT algorithm (Katoh et al. 2009) in Geneious. *Neoascochyta desmazieri* strain CBS 267.69 was included as the outgroup. The sequences of each locus were aligned separately and manually adjusted where necessary.

Maximum likelihood (ML) analysis was run using the RAxML v. 7.2.8 (Stamatakis and Alachiotis 2010) plug-in in Geneious v. 11.1.5 starting from a random tree topology. The nucleotide substitution model used was general time-reversible (GTR) with a gamma-distributed rate variation. The Bayesian analysis was performed using the MrBayes v.3.2.1 (Ronquist and Huelsenbeck 2003) plug-in in Geneious v. 11.1.5. To remove the need for a priori model testing, the Markov chain Monte Carlo (MCMC) analysis was set to sample across the entire GTR model space with a gamma-distributed rate variation across the nucleotide sites. Ten million random trees were generated using the MCMC procedure with four chains. The sample frequency was set at 2000 and the temperature of the heated chain was 0.1. “Burn-in” was set at 25%, after which the log-likelihood values were stationary.

### Morphology

Fungal isolates were cultured on four media types; PDA, oatmeal agar (OA), malt extract agar (MEA) (Boerema et al. 2004; Chen et al. 2015a), and carnation leaf agar (CLA). The colonies were measured at 7 d, and morphology examined after 12–14 d incubation in the same light and temperature conditions described above. Images of the colonies were captured by an Epson Perfection V700 scanner at a 300 dpi resolution. Colony colour was determined on surface and reverse using the colour charts of Rayner (1970). Isolates were characterised microscopically from the PDA plates. Lactic acid (100 % v/v) was used as the mounting fluid. Specimens were examined using a Leica DM5500B compound microscope with a Leica DFC 500 camera fitted to capture images under Nomarski differential interference contrast illumination. Micromorphological measurements and descriptions of pycnidia, pycnidial wall cells and conidia were taken from up to 20 samples, and septation and colour recorded. Images of pycnidia were taken from CLA plates using a Leica M165C stereo microscope and Lecia DFC 500 camera. The NaOH spot test on MEA culture plates helped distinguish taxa (Boerema et al. 2004).

## Results

From 124 samples of legumes collected at 22 locations, 194 isolates were obtained of which 54 isolates were identified as Didymellaceae by ITS sequences. Of these, 36 isolates were further sequenced (*rpb*2 locus). Duplicate isolates were excluded where they were from the same host species, which left 18 isolates for multilocus sequence analysis and inclusion in the phylogenetic analysis.

### Phylogeny

**Figure 1.**
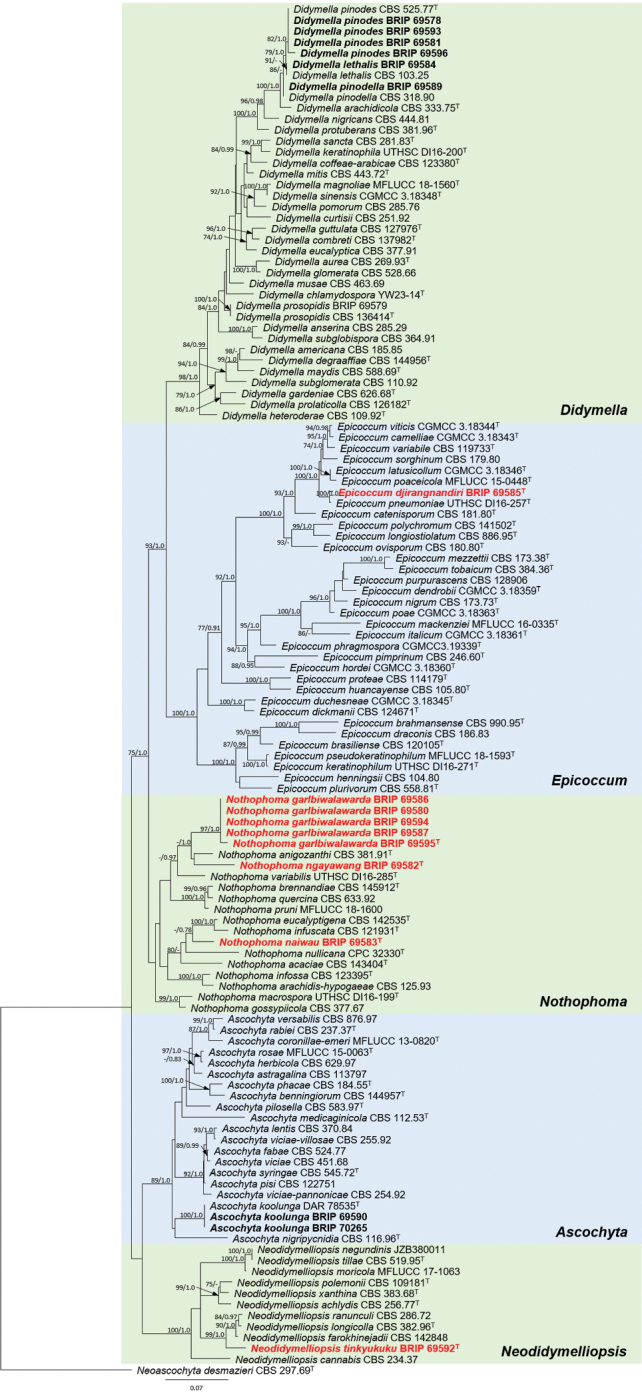
Phylogenetic tree based on maximum likelihood analysis of the combined multilocus (*rpb*2 and ITS) alignment. RAxML bootstrap values (bs) greater than 70 % and Bayesian posterior probabilities (pp) greater than 0.95 are given at the nodes (bs/pp). Genera are delimited in coloured boxes, with the genus name indicated to the right. Isolates identified in this study are in **bold**, and novel taxa are in **red bold**. Ex-type isolates are marked with ^T^. The outgroup is *Neoascochyta desmazieri* (CBS 297.69).

**Figure 2.**
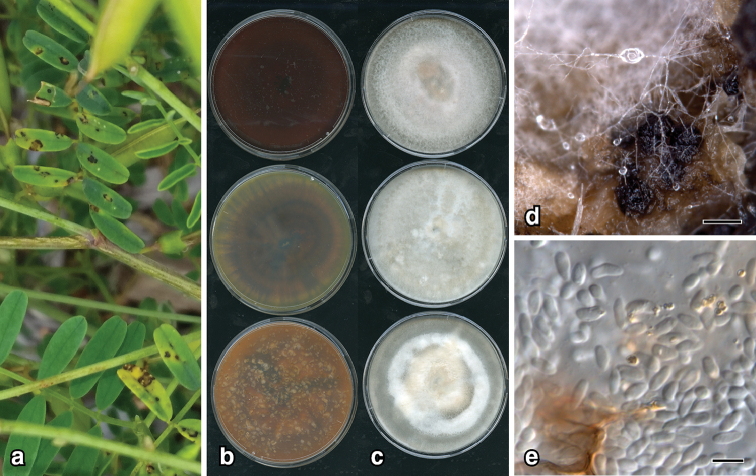
*Epicoccum djirangnandiri*: **a** leaf lesions on *Swainsona galegifolia***b** 14-d old colonies on PDA, MEA, OA (left, top to bottom) and lower surface (right) **c** upper surface **d** pycnidia on CLA**e** conidia. Scale bars: 200 µm (**d**); 7 µm (**e**).

**Figure 3.**
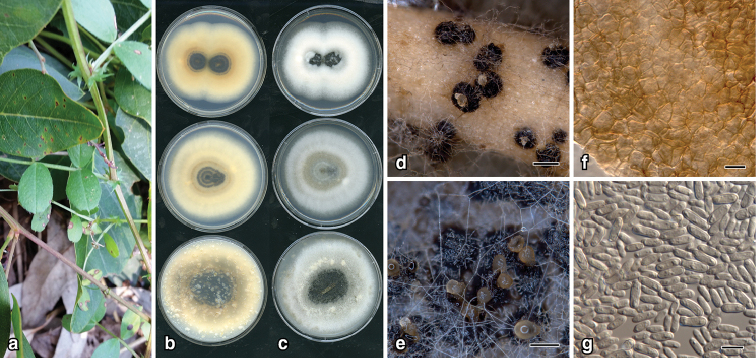
*Neodidymelliopsis tinkyukuku*: **a** leaf lesions on *Hardenbergia violacea***b** 12-d old colonies top to bottom on PDA, MEA, OA (left, top to bottom) and lower surface (right) **c** upper surface **d** pycnidia on CLA**e** pycnidia **f** pycnidial wall **g** conidia. Scale bars: 300 µm (**d, e**); 10 µm (**f**); 7 µm (**g**).

**Figure 4.**
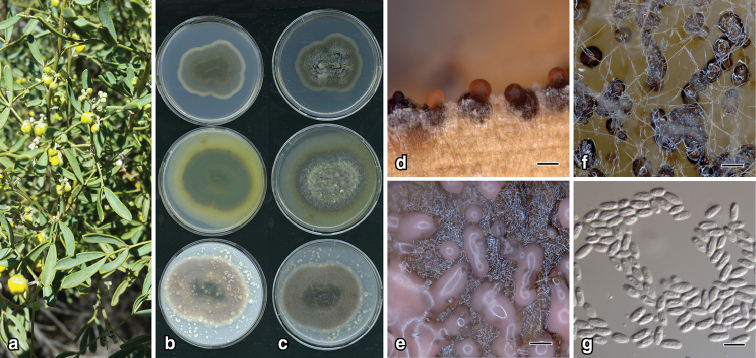
*Nothophoma garlbiwalawarda*: **a** pin-prick leaf spots on *Senna artemisioides* from Wudinna SA**b** 12-d old colonies top to bottom on PDA, MEA, OA (left, top to bottom) and lower surface (right) **c** upper surface **d** pycnidia on CLA**e** pycnidia and pycnidial ooze on OA**f** pycnidia on PDA**g** conidia. Scale bars: 300 µm (**d, e, f**); 7 µm (**g**).

**Figure 5.**
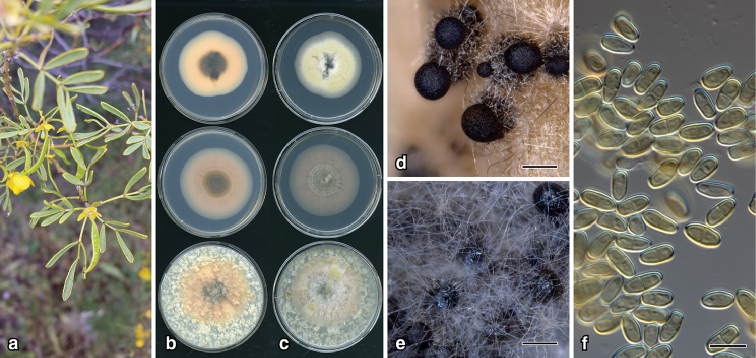
*Nothophoma naiawu*: **a** pin-prick leaf spots on *Senna artemisioides***b** 14-d old colonies top to bottom on PDA, MEA, OA (left, top to bottom) and lower surface (right) **c** upper surface **d** pycnidia on CLA**e** pycnidia **f** conidia. Scale bars: 300 µm (**d, e**); 10 µm (**f)**.

**Figure 6.**
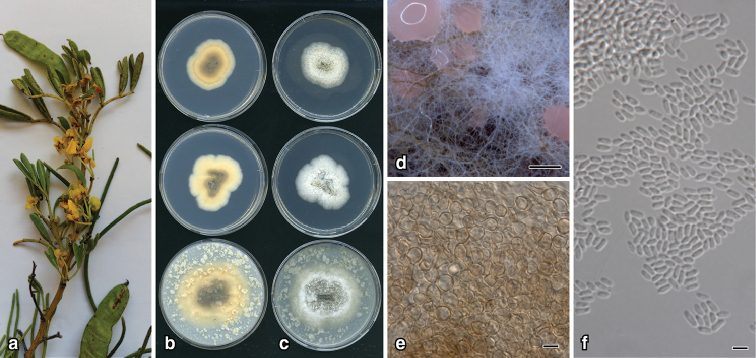
*Nothophoma ngayawang*: **a** leaf and pod lesions on *Senna artemisioides***b** 14-d old colonies, top to bottom on PDA, MEA, OA (left, top to bottom) and lower surface (right) **c** upper surface **d** pycnidia **e** pycnidial wall **f** conidia. Scale bars: 250 µm (**d**); 8 µm (**e**); 3 µm (**f**).

A multilocus sequence analysis based on the ITS region and partial region of the *rpb*2 gene was used to infer the relationship of the 18 isolates and recognised species in Didymellaceae (Table 1). The resulting concatenated aligned dataset comprised 124 ingroup isolates from 111 taxa, and consisted of 1,090 characters (493 for ITS, and 596 for *rpb*2, including alignment gaps). The ML tree based on the combined dataset is presented, with bootstrap support values (BS) greater than 70% and Bayesian posterior probabilities (PP) greater than 0.95 indicating four well-supported clades, and limited support for *Nothophoma* (Fig. 1). The ITS phylogeny, using either ML or Bayesian analysis, provided poor resolution at the genus and species level (data not shown). The phylogenetic tree based on the concatenated alignment of ITS and *rpb*2 indicates the placement of the 18 isolates (Fig. 1), five of which represent novel species (Figs 2–6).

We identified three new host-pathogen associations, and one new record for Australia *Didymella pinodes* (strains BRIP 69581, 69593, and 69596) was isolated from native *S. artemisioides* from three locations in South Australia separated by over 400 km. *Didymella pinodes* (strain BRIP 69578) was also isolated from naturalised *Vicia cracca* (tufted vetch) in New South Wales from an area which did not cultivate *P. sativum*. *Didymella lethalis* (strain BRIP 69584) was isolated from the naturalised *Lathyrus tingitanus* (tangier pea) from a recreational walking area within an urban environment. *Didymella prosopidis* (strain BRIP 69579) was isolated from *Gastrolobium celsianum* from the botanic gardens in the capital city of South Australia, Adelaide.

**Table 1.** Didymellaceae isolates examined in this study. Novel taxa and newly generated sequences are indicated in **bold**.

Species	Strain ^1^	Host	Locality ^2^	GenBank accessions ^3^	
				ITS	*rpb*2
*Ascochyta astragalina*	CBS 113797	*Lathyrus vernus*	Sweden	KT389482	MT018257
*Ascochyta benningiorum*	CBS 144957 ^T^	Soil	The Netherlands	MN823581	MN824606
*Ascochyta coronillae-emeri*	MFLUCC 13-0820 ^T^	*Hippocrepis emerus*	Italy	MH069661	MH069679
*Ascochyta fabae*	CBS 524.77	*Phaseolus vulgaris*	Belgium	GU237880	MT018241
*Ascochyta herbicola*	CBS 629.97	Water	USA, Montana, Missoula	GU237898	KP330421
*Ascochyta koolunga*	DAR 78535 ^T^	*Pisum sativum*	Australia, SA, Minnipa	EU338416	EU874849
	BRIP 70265	*Pisum sativum*	Australia, SA, Riverton	**MN567671**	**MN604922**
	BRIP 69590	*Pisum sativum*	Australia, SA, Mundulla	**MN567672**	**MN604923**
*Ascochyta lentis*	CBS 370.84	*Lens culinaris*	Unknown	KT389474	MT018246
*Ascochyta medicaginicola*	CBS 112.53 ^T^	*Medicago sativa*	USA	GU237749	MT018251
*Ascochyta nigripycnidia*	CBS 116.96 ^T^	*Vicia cracca*	Russia	GU237756	MT018253
*Ascochyta phacae*	CBS 184.55 ^T^	*Phaca alpine*	Switzerland	KT389475	MT018255
*Ascochyta pilosella*	CBS 583.97 ^T^	*Clintonia uniflora*	Canada	MN973590	MT018258
*Ascochyta pisi*	CBS 122785	*Pisum sativum*	The Netherlands	GU237763	MT018244
*Ascochyta rabiei*	CBS 237.37 ^T^	*Cicer arietinum*	Bulgaria	KT389479	MT018256
*Ascochyta rosae*	MFLUCC 15-0063 ^T^	*Rubus ulmifolius*	Italy	KY496751	KY514409
*Ascochyta syringae*	CBS 545.72 ^T^	*Syringa vulgaris*	The Netherlands	KT389483	MT018245
*Ascochyta versabilis*	CBS 876.97	*Silene* sp.	The Netherlands, Wageningen	GU237909	KT389561
*Ascochyta viciae*	CBS 451.68	*Vicia sepium*	The Netherlands, Baarn, Praamgracht	KT389484	KT389562
*Ascochyta viciae-pannonicae*	CBS 254.92	*Vicia pannonica*	Czechoslovakia	KT389485	MT018250
*Ascochyta viciae-villosae*	CBS 255.92	*Vicia villosa*	Czechoslovakia	MN973584	MT018249
*Didymella americana*	CBS 185.85	*Zea mays*	USA, Georgia	FJ426972	KT389594
*Didymella anserina*	CBS 253.80		Germany	KT389498	KT389595
*Didymella arachidicola*	CBS 333 .75 ^T^	*Arachis hypogaea*	South Africa, Cape Province	GU237833	KT389598
*Didymella aurea*	CBS 269.93 ^T^	*Medicago polymorpha*	New Zealand, Auckland	GU237818	KT389599
*Didymella chlamydospora*	YW23-14 ^T^	Soil	South Korea	MK836111	LC480708
*Didymella coffeae-arabicae*	CBS 123380 ^T^	*Coffea Arabica*	Ethiopia	FJ426993	KT389603
*Didymella combreti*	CBS 137982 ^T^	*Combretum mossambiciensis*	Zambia	MN973525	MT018139
*Didymella curtisii*	CBS 251.92	*Nerine* sp.	The Netherlands	FJ427038	MT018131
*Didymella degraaffiae*	CBS 144956 ^T^	Soil	The Netherlands	MN823444	MN824470
*Didymella eucalyptica*	CBS 377.91	*Eucalyptus* sp.	Australia, WA	GU237846	KT389605
*Didymella gardeniae*	CBS 626.68 ^T^	*Gardenia jasminoides*	India	FJ427003	KT389606
*Didymella glomerata*	CBS 528.66	*Chrysanthemum* sp.	The Netherlands	FJ427013	GU371781
*Didymella guttulata*	CBS 127976 ^T^	Soil	Zimbabwe	MN973524	MT018138
*Didymella heteroderae*	CBS 109.92 ^T^	Undefined food material	The Netherlands	FJ426983	KT389601
*Didymella keratinophila*	UTHSC DI16-200 ^T^	*Homo sapiens*	USA	LT592901	LT593039
*Didymella lethalis*	CBS 103.25			GU237729	KT389607
	BRIP 69584	*Lathyrus tingitanus*	Australia, SA, Brownhill Creek	**MN567674**	**MN604925**
*Didymella magnoliae*	MFLUCC 18-1560 ^T^	*Magnolia grandiflora*	China	MK347814	MK434852
*Didymella maydis*	CBS 588.96 ^T^	*Zea mays*	USA, Wisconsin, Hancock	FJ427086	GU371782
*Didymella mitis*	CBS 443.72 ^T^	Soil	South Africa	MN973523	MT018137
*Didymella musae*	CBS 463.69	*Mangifera indica*	India	FJ427026	MT018148
*Didymella nigricans*	CBS 444.81	*Acer palmatum*	Japan	KY742075	KY742158
*Didymella pinodella*	CBS 318.90	*Pisum sativum*	The Netherlands	FJ427051	MN983533
	BRIP 69589	*Pisum sativum*	Australia, VIC, Rainbow	**MN567675**	**MN604926**
*Didymella pinodes*	CBS 525.77 ^T^	*Pisum sativum*	Belgium	GU237883	KT389614
	BRIP 69581	*Senna artemisioides*	Australia, SA, Blanchetown	**MN567676**	**MN604927**
	BRIP 69593	*Senna artemisioides*	Australia, SA, Blyth	**MN567677**	**MN604928**
	BRIP 69596	*Senna artemisioides*	Australia, SA, Wudinna	**MN567678**	**MN604929**
	BRIP 69578	*Vicia cracca*	Australia, NSW, Cowra	**MN567679**	**MN604930**
*Didymella pomorum*	CBS 539.66	*Polygonum tataricum*	The Netherlands	FJ427056	KT389618
*Didymella prolaticolla*	CBS 126182 ^T^	Soil	Namibia	MN973533	MT018157
*Didymella prosopidis*	CBS 136414 ^T^	*Prosopis* sp.	South Africa	KF777180	MT018149
	BRIP 69579	*Gastrolobium celsianum*	Australia, SA, Adelaide	**MN5676780**	**MN604931**
*Didymella protuberans*	CBS 381.96 ^T^	*Lycium halifolium*	The Netherlands	GU237853	KT389620
*Didymella sancta*	CBS 281.83 ^T^	*Ailanthus altissima*	South Africa	FJ427063	KT389623
*Didymella sinensis*	CGMCC 3.18348 ^T^	*Cerasus pseudocerasus*	China	KY742085	MT018127
*Didymella subglobispora*	CBS 364.91 ^T^	*Ananas sativus*		MN973531	MT018153
*Didymella subglomerata*	CBS 110.92	*Triticum* sp.	USA, North Dakota	FJ427080	KT389626
*Epicoccum brahmansense*	CBS 990.95 ^T^	Soil	Papua New Guinea	MN973513	MT018119
*Epicoccum brasiliense*	CBS 120105 ^T^	*Amaranthus* sp.	Brazil	GU237760	KT389627
*Epicoccum camelliae*	CGMCC 3.18343 ^T^	*Camellia sinensis*	China	KY742091	KY742170
*Epicoccum catenisporum*	CBS 181.80 ^T^	*Oryza sativa*	Guinea-Bissau	FJ427069	LT623253
*Epicoccum dendrobii*	CGMCC 3.18359 ^T^	*Dendrobium fimbriatum*	China	KY742093	MT018084
*Epicoccum dickmanii*	CBS 124671 ^T^	*Acropora Formosa*	Australia	MN973509	MT018113
***Epicoccum djirangnandiri* sp. nov.**	BRIP 69585 ^T^	*Swainsona galegifolia*	Australia, NSW, Mount Annan	**MN567673**	**MN604924**
*Epicoccum draconis*	CBS 186.83	*Dracaena* sp.	Rwanda	GU237795	KT389628
*Epicoccum duchesneae*	CGMCC 3.18345 ^T^	*Duchesnea indica*	China	KY742095	MT018115
*Epicoccum henningsii*	CBS 104.80	*Acacia mearnsii*	Kenya	GU237731	KT389629
*Epicoccum hordei*	CGMCC 3.18360 ^T^	*Hordeum vulgare*	Australia	KY742097	MT018102
*Epicoccum huancayense*	CBS 105.80 ^T^	*Solanum* sp.	Peru	GU237732	KT389630
*Epicoccum italicum*	CGMCC 3.18361 ^T^	*Acca sellowiana*	Italy	KY742099	KY742172
*Epicoccum keratinophilum*	UTHSC DI16-271 ^T^	*Homo sapiens*	USA	LT592930	LT593068
*Epicoccum latusicollum*	CGMCC 3.18346 ^T^	*Sorghum bicolor*	China	KY742101	KY742174
*Epicoccum longiostiolatum*	CBS 886.95 ^T^	*Stellaria* sp.	Papua New Guinea	FJ427074	MT018108
*Epicoccum mackenziei*	MFLUCC 16-0335 ^T^	*Ononis spinose*	Italy	KX698039	KX698035
*Epicoccum mezzettii*	CBS 173.38 ^T^	*Populus* pulp	Italy	MN973496	MT018095
*Epicoccum nigrum*	CBS 173.73 ^T^	*Dactylis glomerata*	USA	FJ426996	KT389632
*Epicoccum ovisporum*	CBS 180.80 ^T^	*Zea mays*	South Africa	FJ427068	LT623252
*Epicoccum phragmospora*	CGMCC 3.19339 ^T^	*Saccharum officinarum*	China	MN215619	MN255460
*Epicoccum pimprinum*	CBS 246.60 ^T^	Soil	India	FJ427049	MT018100
*Epicoccum plurivorum*	CBS 558.81 ^T^	*Setaria* sp.	New Zealand	GU237888	KT389634
*Epicoccum pneumoniae*	UTHSC DI16-257 ^T^	*Homo sapiens*	USA	LT592927	LT593065
*Epicoccum poaceicola*	MFLUCC 15-0448 ^T^	Poaceae	Thailand	KX965727	KX898365
*Epicoccum poae*	CGMCC 3.18363 ^T^	*Poa annua*	USA	KY742113	KY742182
*Epicoccum polychromum*	CBS 141502 ^T^	*Paspalum dilinateum*	France	MN973506	MT018109
*Epicoccum proteae*	CBS 114179 ^T^	*Protea compacta* x *Protea neriifolia*	South Africa, Somerset West	JQ044433	LT623251
*Epicoccum pseudokeratinophilum*	MFLUCC 18-1593 ^T^	*Prunus avium*	China	MH827002	MH853659
*Epicoccum purpurascens*	CBS 128906	Soil	USA	MN973488	MT018083
*Epicoccum sorghinum*	CBS 179.80	*Sorghum bicolor*	Puerto Rico	FJ427067	KT389635
*Epicoccum tobaicum*	CBS 384.36 ^T^	Soil	Indonesia	MN973493	MT018092
*Epicoccum variabile*	CBS 119733 ^T^	*Coffea Arabica*	Brazil	MN973501	MT018103
*Epicoccum viticis*	CGMCC 3.18344 ^T^	*Vitex negundo*	China	KY742118	KY742186
*Neoascochyta desmazieri* (outgroup)	CBS 297.69 ^T^	*Lolium perenne*	Germany, Hohenlieth	KT389508	KT389644
*Neodidymelliopsis achlydis*	CBS 256.77 ^T^	*Achlys triphylla*	Canada, British Columbia, Vancouver Island	KT389531	MT018293
*Neodidymelliopsis cannabis*	CBS 234.37	*Cannabis sativa*	Unknown	GU237804	KP330403
*Neodidymelliopsis farokhinejadii*	CBS 142853	*Conocarpus erectus*	Iran	KY449009	KY464922
*Neodidymelliopsis longicolla*	CBS 382.96 ^T^	Soil	Israel, En Avdat, Negev desert	KT389532	MT018298
*Neodidymelliopsis moricola*	MFLUCC 17-1063	*Morus alba*	Russia	KY684939	KY684943
*Neodidymelliopsis negundinis*	JZB380011	*Acer negundo*	Russia	MG564165	MG564166
*Neodidymelliopsis polemonii*	CBS 109181 ^T^	*Polemonium caeruleum*	The Netherlands	GU237746	KP330427
*Neodidymelliopsis ranunculi*	CBS 286.72	*Citrus limonium*	Italy	MN973612	MT018294
*Neodidymelliopsis tillae*	CBS 519.95 ^T^	*Tilia* sp.	Italy	MN973610	MT018287
***Neodidymelliopsis tinkyukuku* sp. nov.**	BRIP 69592 ^T^	*Hardenbergia violacea*	Australia, SA, Clare	**MN5676781**	**MN604932**
*Neodidymelliopsis xanthina*	CBS 383.68 ^T^	*Delphinium* sp.	The Netherlands, Baarn	GU237855	KP330431
*Nothophoma acaciae*	CBS 143404 ^T^	*Acacia melanoxylon*	Australia	MG386056	MG386144
*Nothophoma anigozanthi*	CBS 381.91 ^T^	*Anigozanthus maugleisii*	The Netherlands	GU237852	KT389655
*Nothophoma arachidis-hypogaeae*	CBS 125.93	*Arachis hypogaea*	India, Madras	GU237771	KT389656
*Nothophoma brennandiae*	CBS 145912 ^T^	Soil	The Netherlands	MN823579	MN824604
***Nothophoma garlbiwalawarda* sp. nov.**	BRIP 69580	*Senna artemisioides*	Australia, SA, Adelaide	**MN5676782**	**MN604933**
	BRIP 69586	*Senna artemisioides*	Australia, SA, Berri	**MN5676783**	**MN604934**
***Nothophoma garlbiwalawarda* sp. nov.**	BRIP 69587	*Senna artemisioides*	Australia, SA, Berri	**MN5676784**	**MN604935**
	BRIP 69594	*Senna artemisioides*	Australia, SA, Kimba	**MN5676785**	**MN604936**
	BRIP 69595 ^T^	*Senna artemisioides*	Australia, SA, Wudinna	**MN5676786**	**MN604937**
*Nothophoma eucalyptigena*	CBS 142535 ^T^	*Eucalyptus* sp.	Australia	KY979771	KY979852
*Nothophoma gossypiicola*	CBS 377.67	*Gossypium* sp.	USA, Texas	GU237845	KT389658
*Nothophoma infossa*	CBS 123395 ^T^	*Fraxinus pennsylvanica*	Argentina, Buenos Aires Province, La Plata	FJ427025	KT389659
*Nothophoma infuscata*	CBS 121931 ^T^	*Acacia longifolia*	New Zealand	MN973559	MN973559
*Nothophoma macrospora*	UTHSC DI16-199 ^T^	*Homo sapiens*	USA, Arizona	LN880536	LT593073
***Nothophoma naiawu* sp. nov.**	BRIP 69583 ^T^	*Senna artemisioides*	Australia, SA, Blanchetown	**MN5676787**	**MN604938**
	BRIP 69582 ^T^	*Senna artemisioides*	Australia, SA, Blanchetown	**MN5676788**	**MN604939**
*Nothophoma nullicana*	CPC 32330 ^T^	*Acacia falciformis*	Australia	NR_156665	MG386143
*Nothophoma pruni*	MFLUCC 18-1600	*Prunus avium*	China	MH827005	MH853662
*Nothophoma quercina*	CBS 633.92	*Microsphaera alphitoides* from *Quercus* sp.	Ukraine	GU237900	KT389657
*Nothophoma variabilis*	UTHSC DI16-285 ^T^	*Homo sapiens*	USA	LT592939	LT593078

^1^BRIP, Queensland Plant Pathology Herbarium, Brisbane, QLD, Australia; CBS, Westerdijk Fungal Biodiversity Institute, Utrecht, the Netherlands; CGMCC, China General Microbiological Culture Collection, Beijing, China; MFLUCC, Mae Fah Luang University Culture Collection, Chiang Rai, Thailand; UTHSC, Fungus Testing Laboratory at the University of Texas Health Science Center, San Antonio, Texas, USA. ^2^NSW, New South Wales; SA, South Australia; VIC, Victoria; WA, Western Australia. ^3^ITS, internal transcribed spacer region; *rpb*2, RNA polymerase II second subunit. ^T^ ex-type strain.

### Taxonomy

Multilocus sequence analysis and morphological comparisons classified nine fungal isolates from legumes in southern Australia into five novel species from three Didymellaceae genera. The novel species are described and illustrated in Figs 2–6. Nomenclatural novelties are registered in MycoBank.

The species epithets were derived from Indigenous Australian Peoples’ language groups to provide a uniquely Australian theme. Permission to use words from the local language of the area in which the fungi were collected was granted by elders or community representatives.

#### Epicoccum djirangnandiri

Taxon classificationFungiPleosporalesDidymellaceae

E.C. Keirnan, M.H. Laurence, R.G. Shivas & Y.P. Tan sp. nov.

215FEA5D-B1DB-576B-85B6-6BF8A79E7087

833689 Fig. 2

##### Type.

Australia, New South Wales, Mount Annan, *Swainsona galegifolia*, 19 Jan. 2017, *E.C. Keirnan* (holotype BRIP 69585, includes culture ex-type).

##### Description.

*Colonies* on OA, 76–80 mm diam. after 7 d, covered in dense aerial mycelium, variable shades of grey, pale cinnamon towards centre; reverse dark vinaceous; on MEA, 70–72 mm after 7 d, margin entire, covered in low dense aerial mycelium, pale mouse grey with lighter patches; reverse olivaceous with radiating spokes; on PDA, 73–80 mm after 7 d, margin entire, mycelia felty, mouse grey becoming vinaceous buff towards centre; reverse fuscous black. *NaOH spot test*: negative. *Conidiomata* on CLA, pycnidial, globose 100–200 μm diam., pale brown becoming black, solitary, glabrous, non-papillate; pycnidial wall composed of textura globulosa, pale brown, cells 5–15 μm diam. *Conidiogenous* cells phialidic, cylindral, thin-walled, hyaline, rounded ends. *Conidia* aseptate, 5–7 × 2–3 μm.

##### Etymology.

From the language of the Indigenous Australian Dharawal people, meaning leaf spot. The Dharawal people are from the western Sydney region in New South Wales, which includes Mount Annan, where the holotype was collected.

##### Notes.

*Epicoccum djirangnandiri* is phylogenetically close to *E. pneumoniae* ex-type strain UTHSC DI16-257 (Fig. 1) and is distinguished in *rpb*2 sequences with 99% identity. Morphological comparisons could not be made as *E. pneumoniae* was sterile in culture (Valenzuela-Lopez et al. 2018). *Epicoccum djirangnandiri* is only known from one specimen on *Swainsona galegifolia*.

#### Neodidymelliopsis tinkyukuku

Taxon classificationFungiPleosporalesDidymellaceae

E.C. Keirnan, M.H. Laurence, R.G. Shivas & Y.P. Tan sp. nov.

99FBBB09-AB5C-588E-9815-F1D246805CCA

833692

Fig. 3

##### Type.

Australia, South Australia, Clare, *Hardenbergia violacea*, 17 Sep. 2017, *E.C. Keirnan* (holotype BRIP 69592, includes culture ex-type).

##### Description.

*Colonies* on OA, 26–28 mm diam. after 7 d, dense low aerial mycelium, buff with numerous grey patches, darker with abundant pycnidia at centre; reverse buff to rosy buff with darker concentric rings towards centre; on MEA, 28–30 mm after 7 d, margin entire, dense low aerial mycelium, vinaceous buff paler at margin; reverse rosy buff to buff at margin with abundant scattered pycnidia; on PDA, 35–38 mm after 7 d, margin entire, dense low aerial mycelium, pale mouse grey lighter at margin; reverse cinnamon with concentric dark rings, darker at centre. *NaOH spot test*: light yellow. *Conidiomata* on CLA pycnidial, globose to ampulliform, 250–350 μm diam., brown becoming black, solitary, abundant in centre of colony, zonate, glabrous, non-papillate; ostiole c. 25 μm diam.; pycnidial wall composed of textura angularus, pale brown, cells 5–8 μm diam. *Conidiogenous cells* phialidic, cylindrical, thin-walled, hyaline. *Conidia* occasionally septate, 6–9 × 2–3 μm, cylindrical, hyaline, thin-walled.

##### Etymology.

From the language of the Indigenous Australian Kaurna people, meaning leaf disease. The Kaurna people are from the Adelaide plains region, which includes Clare, the locality where the holotype was collected.

##### Notes.

*Neodidymelliopsis tinkyukuku* (strain BRIP 69592) is sister to a clade that includes *N. farokhinejadii* (strain CBS 142853), *N. longicolla* (ex-type strain CBS 382.96) and *N. ranunculi* (strain CBS 286.72) (Fig. 1). *Neodidymelliopsis* conidial dimensions are distinct from *N. farokhinejadii* (4.6–7.5 × 2.4–3.9 μm), *N. longicolla* (12–15 × 4–7 μm), and *N. ranunculi* (3–5 × 7.5–10 μm). *Neodidymelliopsis tinkyukuku* can be easily distinguished from these three species by DNA sequences of the *rpb2* locus.

#### Nothophoma garlbiwalawarda

Taxon classificationFungiPleosporalesDidymellaceae

E.C. Keirnan, M.H. Laurence, R.G. Shivas & Y.P. Tan sp. nov.

1CA11AC0-D737-5DA2-9F63-DDFFEA13D2AA

833693

Fig. 4

##### Type.

Australia, South Australia, Wudinna, *Senna artemisioides*, 19 Aug. 2017, *E.C. Keirnan* (holotype BRIP 69595, includes culture ex-type).

##### Description.

*Colonies* on OA, 27–30 mm diam. after 7 d, flat with scant aerial mycelia with a few zonate rings, vinaceous to dark vinaceous; vinaceous to dark vinaceous; on MEA, 23–25 mm after 7 d, margin entire, flat, scant aerial mycelium towards centre, amber with abundant pycnidia; reverse amber darker towards centre; on PDA, 28–30 mm after 7 d, margin irregular, flat with aerial mycelia tufted in centre, dark with abundant pycnidia in concentric rings, buff at margin; reverse dark becoming buff at margin. *NaOH spot test*: reddish. *Conidiomata* pycnidial, globose to subglobose, 130–320 μm diam., pale brown, scattered, abundant, zonate, glabrous, non-papillate; ostiole c. 25 μm diam.; pycnidial wall composed of textura angularus, pale to medium brown, cells 5–12 μm diam. *Conidiogenous* cells phialidic, cylindrical, thin-walled, hyaline 5–12 × 2–4 μm long, narrower at the apex. *Conidia* aseptate, 5–7.0 × 2.0–3.0 μm, parallel to narrowly ellipsoidal, hyaline, wall c. 0.5 μm.

##### Etymology.

From the native language of the Indigenous Australian Barngarla people, meaning leaf-fun-guy. The Barngarla people are from the Eyre Peninsula region, which includes Wudinna, the locality where the holotype was collected.

##### Additional material examined.

Australia, South Australia, Adelaide, *Senna artemisioides*, 26 Oct. 2016, *E.C. Keirnan* (BRIP 69580); Berri, *Senna artemisioides*, 01 Jul. 2017, *E.C. Keirnan* (BRIP 69586); ibid, 01 Jul. 2017, *E.C. Keirnan* (BRIP 69587); Kimba, *Senna artemisioides*, 17 Sep. 2017, *E.C. Keirnan* (BRIP 69594).

##### Notes.

*Nothophoma garlbiwalawarda* is phylogenetically closest to *No. anigozanthi* and two novel species (see below for notes) (Fig. 2). *Nothophoma garlbiwalawarda* is distinguished from *No. anigozanthi* by its larger conidia (cf. 3.5–5 × 1.5–2.5 μm), *rpb2* sequence (93% identity), and its reaction to *NaOH spot test* on MEA (dull green then black).

#### Nothophoma naiawu

Taxon classificationFungiPleosporalesDidymellaceae

E.C. Keirnan, M.H. Laurence, R.G. Shivas & Y.P. Tan sp. nov.

4045D173-B744-5746-9924-46212C012F80

833694

Fig. 5

##### Type.

Australia, South Australia, Blanchetown, from *Senna artemisioides*, 22 Oct. 2016, *E.C. Keirnan*, holotype BRIP 69583 (includes culture ex-type).

##### Description.

*Colonies* on OA, 21–25 mm diam. after 7 d, flat with scant aerial mycelia, rosy vinaceous, dark at centre; reverse rosy buff, dark at centre, with a few dark radiating fissures; on MEA, 27–30 mm after 7 d, margin entire, flat, with sparse aerial mycelium towards centre rosy vinaceous; reverse peach, darker at centre; on PDA, 27–30 mm after 7 d, margin entire, flat felty, rosy buff; reverse peach, dark at centre. *NaOH spot test*: slightly yellow. *Conidiomata* pycnidial, globose to subglobose, 200–300 μm diam., pale brown becoming black, semi-immersed, confluent on MEA, glabrous, non-papillate; ostiole c. 25 μm diam.; pycnidial wall composed of textura globulosa, pale brown, cells 5–8 μm diam.. *Conidiogenous* cells phialidic, cylindrical, very thin-walled, hyaline. *Conidia* aseptate or 1-septate, 8–12 × 4–6 μm, cylindrical to narrow ellipsoidal, pale yellow.

##### Etymology.

A variation of the Indigenous Australian Ngayawang people’s language group, who lived in the Murray River region of South Australia, which includes Blanchetown, the locality where this specimen was collected.

##### Notes.

*Nothophoma naiawu* is phylogenetically close to *No. eucalyptigena* and *No. infuscata* (Fig. 2). *Nothophoma naiawu* is easily distinguished from *No. eucalyptigena* and *No. infuscata* by the ITS region (98 % identity to both) and the *rpb*2 locus (95%, and 94% identity, respectively). *Nothophoma infuscata* produce a pale red discolouration in response to *NaOH spot test* on MEA media, which is distinct from the slightly yellow response by *No. naiawu*.

#### Nothophoma ngayawang

Taxon classificationFungiPleosporalesDidymellaceae

E.C. Keirnan, M.H. Laurence, R.G. Shivas & Y.P. Tan sp. nov.

A9352891-7546-5110-9BB1-05B9446D1859

833695

Fig. 6

##### Type.

Australia, South Australia, Blanchetown, *Senna artemisioides*, 22 Oct. 2016, *E.C. Keirnan*, holotype BRIP 69582 (includes culture ex-type).

##### Description.

*Colonies* on OA, 18–20 mm diam. after 7 d, covered by scant tufted aerial mycelia at centre becoming abundant and floccose towards margin, rosy buff becoming darker towards centre; reverse salmon with centre and margins pale isabelline; on MEA, 15–20 mm after 7 d, margin irregular, felty buff becoming white towards the margin; reverse pale rosy buff, darker at centre becoming paler near margin; on PDA, 18–21 mm after 7 d, margin regular, aerial mycelia tufted in centre becoming floccose toward the margin, white to pale rosy buff; reverse pale rosy buff with few scattered vinaceous spots. *NaOH spot test*: slightly yellow. *Conidiomata* pycnidial, globose to subglobose, 200–300 μm diam., pale brown becoming black, solitary, abundant in centre of colony, glabrous, non-papillate; ostiole c. 25 μm diam.; pycnidial wall composed of textura globulosa, pale brown, cells 5–8 μm diam. *Conidiogenous* cells phialidic, cylindrical, thin-walled, hyaline. *Conidia* aseptate, 2.5–4.0 × 1.0–2.0 μm, cylindrical to narrow ellipsoidal, hyaline, thin-walled.

##### Etymology.

Named after the Indigenous Australian Ngayawang people’s language group, who existed in the Murray River region of South Australia, which includes Blanchetown, the locality where this specimen was collected.

##### Notes.

*Nothophoma ngayawang* is phylogenetically close to *No. anigozanthi* ex-type strain CBS 381.91 (Fig. 2). *Nothophoma ngayawang* is distinguished from *No. variabilis* by the ITS region (98 % identity) and the *rpb*2 locus (93% identity). The *NaOH spot test* of *No. variabilis* was negative on MEA, which is distinguished from the slightly yellow reaction of *No. ngayawang*.

## Discussion

Our investigations did not identify *A. koolunga* from native Australian legumes. In fact, the incidence was low in that only one isolate (BRIP 69590) was collected from *P. sativum* in South Australia. It is difficult to make an association between the low incidence of *A. koolunga* on *P. sativum* and the absence of *A. koolunga* on other legumes. While the current evidence suggests that *A. koolunga* is unlikely to have originated from Australian native legumes, additional field surveys may be required to investigate the possible source of *A. koolunga*.

Our investigations instead uncovered five novel Didymellaceae species not yet known to science. *Epicoccum djirangnandiri* on *S. galegifolia* was collected from the botanic garden in New South Wales, where the host is endemic. *Neodidymelliopsis tinkyukuku* on *H. violacea* was collected from a public garden in South Australia. Growing in the same garden is *V. sativa* from which *D. pinodes* (strain BRIP 69578), a known Ascochyta blight pathogen, was isolated. *Hardenbergia violacea* has a wide distribution in southern and eastern Australia. These three native Australian legume species were found in a cultivated environment rather than in a natural environment. Further studies are warranted to understand how widespread these fungal species may be in cultivated or natural environments, and if they are host specific.

Leaf spots were commonly seen on the native legume *S. artemisioides* throughout the regions sampled in South Australia. Three novel *Nothophoma* species were isolated from *S. artemisioides*. *Nothophoma garlbiwalawarda* was collected from five locations across South Australia, separated by over 400 km, in field pea and non-field pea growing regions. *Nothophoma naiawu* and *No. ngayawang* were collected from the South Australian Murray River region on the roadside of a main highway. The leaf spot symptoms for the three *Nothophoma* species were similar (small pin-prick lesions), with some larger spots on the seed pods caused by *No. ngayawang*.

Our investigations also identified new host-pathogen associations, namely *D. pinodes* on *S. artemisioides* and *V. cracca*, and *D. lethalis* on *L. tingitanus*. These hosts could be a reservoir of Ascochyta blight inoculum if found growing adjacent to field pea crops. The discovery of an alternative host has implications for disease epidemiology and management. The symptoms of *D. pinodes* on *S. artemisioides* are indistinguishable from the pin-prick leaf spot symptoms caused by the three *Nothophoma* species described in this study. *Didymella pinodes* was isolated from five locations. Four of these locations also yielded a novel *Nothophoma* species. *Didymella prosopidis* was isolated from the Australian native *G. celsianum*, a species first described as associated with stem disease of *Prosopis* sp. (also a member of the Fabaceae family) in South Africa (Crous et al. 2013). This is the first report of *D. prosopidis* outside of South Africa.

At the outset, our study sought to identify if any *A. koolunga* could be isolated from Australian native legumes causing leaf spot disease. This study uncovered five novel isolates in the Didymellaceae from Australian native legumes, and identified three new legume host-pathogen associations for Australia. *Ascochyta koolunga* was not isolated from hosts other than field pea, which might be an artefact of the low incidence of the fungus during the collection period. Further investigations using a longitudinal systematic survey are needed to identify any native hosts of *A. koolunga* and to further investigate the diversity and prevalence of Didymellaceae species on Australian native, pasture and naturalised legumes, to classify novel isolates and to identify new Australian hosts for known species.

## Supplementary Material

XML Treatment for Epicoccum djirangnandiri

XML Treatment for Neodidymelliopsis tinkyukuku

XML Treatment for Nothophoma garlbiwalawarda

XML Treatment for Nothophoma naiawu

XML Treatment for Nothophoma ngayawang

## References

[B1] Ahmed H Chang K-F Hwang S-F Fu H Zhou Q Strelkov S Conner R Gossen B (2015) Morphological characterization of fungi associated with the ascochyta blight complex and pathogenic variability of *Mycosphaerella pinodes* on field pea crops in central Alberta. The Crop Journal 3: 10–18. 10.1016/j.cj.2014.08.007

[B2] Ali SM Dennis J (1992) Host range and physiologic specialisation of *Macrophomina phaseolina* isolated from field peas in South Australia. Jounal of Experimental Agriculture 32: 1121–1125. 10.1071/EA9921121

[B3] Ariyawansa HA Hyde KD Jayasiri SC (2015) Fungal diversity notes 111–252–taxonomic and phylogenetic contributions to fungal taxa. Fungal Diversity 75: 27–274. 10.1007/s13225-015-0346-5

[B4] Aveskamp MM Verkley GJM de Gruyter J Murace MA Perello A Woudenberg JHC Groenewald JZ Crous PW (2009) DNA phylogeny reveals polyphyly of Phoma section Peyronellaea and multiple taxonomic novelties. Mycologia 101: 363–382. 10.3852/08-199 19537209

[B5] Aveskamp MM de Gruyter J Woudenberg JH Verkley GJ Crous PW (2010) Highlights of the *Didymellaceae*: A polyphasic approach to characterise *Phoma* and related pleosporalean genera. Studies in Mycology 65: 1–60. 10.3114/sim.2010.65.01 20502538 PMC2836210

[B6] Boerema GH De Gruyter J Noordeloos ME Hamers MCE (2004) *Phoma* identifiction manual differention of specific and intra-specific taxa in culture. CABI Publishing, Cambridge, MA, USA, Wallingford, OX, UK, 10.1079/9780851997438.0000

[B7] Chen Q Jiang JR Zhang GZ Crous PW (2015a) Resolving the *Phoma* enigma. Studies in Mycology 82: 137–217. 10.1016/j.simyco.2015.10.003 26955202 PMC4774273

[B8] Chen Q Zhang KE Zhang G Cai L (2015b) A polyphasic approach to characterise two novel species of Phoma (Didymellaceae) from China. Phytotaxa 197: 267–281. 10.11646/phytotaxa.197.4.4

[B9] Chen Q Hou LW Duan WJ Crous PW Cai L (2017) *Didymellaceae* revisited. Studies in Mycology 87: 105–159. 10.1016/j.simyco.2017.06.002 28706324 PMC5498420

[B10] Chilvers MI Rogers JD Dugan FM Stewart JE Chen W Peever TL (2009) *Didymella pisi* sp. nov., the teleomorph of *Ascochyta pisi*. Mycological Research 113: 391–400. 10.1016/j.mycres.2008.11.017 19116165

[B11] Crous PW Wingfield MJ Guarro J Cheewangkoon R van der Bank M Swart WJ Stchigel AM Cano-Lira JF Roux J Madrid H Damm U Wood AR Shuttleworth LA Hodges CS Munster M de Jesús Yáñez-Morales M Zúñiga-Estrada L Cruywagen EM de Hoog GS Silvera C Najafzadeh J Davison EM Davison PJ Barrett MD Barrett RL Manamgoda DS Minnis AM Kleczewski NM Flory SL Castlebury LA Clay K Hyde KD Maússe-Sitoe SN Chen S Lechat C Hairaud M Lesage-Meessen L Pawłowska J Wilk M Sliwińska-Wyrzychowska A Mętrak M Wrzosek M Pavlic-Zupanc D Maleme HM Slippers B Mac Cormack WP Archuby DI Grünwald NJ Tellería MT Dueñas M Martín MP Marincowitz S de Beer ZW Perez CA Gené J Marin-Felix Y Groenewald JZ (2013b) Fungal Planet description sheets: 154–213. Persoonia 31: 188–296. 10.3767/003158513X675925 24761043 PMC3904050

[B12] Crous PW Groenewald JZ (2016) They seldom occur alone. Fungal Biology 120: 1392–1415. 10.1016/j.funbio.2016.05.009 27742096

[B13] Das K Lee S-Y Jung H-Y (2020) Molecular and morphological characterization of two novel species collected from Soil in Korea. Mycobiology 48:1, 9–19. 10.1080/12298093.2019.1695717 PMC7048220 32158601

[B14] Davidson JA Hartley D Priest M Krysinska-Kaczmarek M Herdina McKay A Scott ES (2009) A new species of *Phoma* causes ascochyta blight symptoms on field peas (*Pisum sativum*) in South Australia. Mycologia 101: 120–128. 10.3852/07-199 19271674

[B15] Davidson JA Krysinska-Kaczmarek M Wilmshurst CJ McKay A Herdina Scott ES (2011) Distribution and survival of ascochyta blight pathogens in field-pea-cropping soils of Australia. Plant Disease 95: 1217–1223. 10.1094/PDIS-01-11-0077 30731696

[B16] Dear S Staden R (1992) A standard file format for data from DNA sequencing instruments. DNA Sequence. 3: 107–110. 10.3109/10425179209034003 1457811

[B17] de Gruyter J Aveskamp MM Woudenberg JH Verkley GJ Groenewald JZ Crous PW (2009) Molecular phylogeny of *Phoma* and allied anamorph genera: towards a reclassification of the *Phoma* complex. Mycological Research 113: 508–519. 10.1016/j.mycres.2009.01.002 19272327

[B18] de Gruyter J (2012) Revised taxonomy of *Phoma* and allied genera. PhD Dissertation, Wageningen University, Wageningen, NL, 181 pp.

[B19] Gaurilcikiene I Viciene RC (2013) The susceptibility of pea (*Pisum sativum* L.) to ascochyta blight under Lithuanian conditions. Zemdirbyste (Agriculture) 100: 283–288. 10.13080/z-a.2013.100.036

[B20] Hibbett D Abarenkov K Koljalg U Opik M Chai B Cole JR Wang Q Crous PW Robert VA Helgason T Herr J Kirk P Lueschow S O’Donnell K Nilsson H Oono R Schoch CL Smyth C Walker D Porras-Alfaro A Taylor JW Geiser DM (2016) Sequence-based classification and identification of Fungi. Mycologia 108: 1049–1068. 27760854 10.3852/16-130

[B21] Hou LW Groenewald JZ Pfenning LH Yarden O Crous PW Cai L (2020) The phoma-like dilemma. Studies in Mycology 96: 309–396. 10.1016/j.simyco.2020.05.001 32904212 PMC7452269

[B22] Katoh K Asimenos G Toh H (2009) Multiple alignment of DNA sequences with MAFFT. In: Posada D (Ed.) Bioinformatics for DNA Sequence Analysis.Humana Press, New York, NY 10013, USA, 39–64. 10.1007/978-1-59745-251-9_3 19378139

[B23] Le May C Potage G Andrivon D Tivoli B Outreman Y (2009) Plant disease complex: Antagonism and synergism between pathogens of the Ascochyta blight complex on pea. Journal of Phytopathology 157: 715–721. 10.1111/j.1439-0434.2009.01546.x

[B24] Liu J Cao T Feng J Chang K-F Hwang S-F Strelkov SE (2013) Characterization of the fungi associated with ascochyta blight of field pea in Alberta, Canada. Crop Protection 54: 55–64. 10.1016/j.cropro.2013.07.016

[B25] Liu N Xu S Yao X Zhang G Mao W Hu Q Feng Z Gong Y (2016) Studies on the Control of Ascochyta Blight in Field Peas (*Pisum sativum* L.) Caused by *Ascochyta pinodes* in Zhejiang Province, China. Frontiers in Microbiology 7: 481–453. 10.3389/fmicb.2016.00481 27148177 PMC4828446

[B26] Liu YJ Whelen S Hall BD (1999) Phylogenetic relationships among ascomycetes: evidence from an RNA polymerase II subunit. Molecular Biology and Evolution 16: 1799–1808. 10.1093/oxfordjournals.molbev.a026092 10605121

[B27] Mathew FM Goswami RS Markell SG Osborne L Tande C Ruden B (2010) First report of Ascochyta blight of field pea caused by *Ascochyta pisi* in South Dakota. Plant Disease 94: 789. 10.1094/PDIS-94-6-0789A 30754333

[B28] O’Donnell K Sarver BAJ Brandt M Chang DC Noble-Wang J Park BJ Sutton DA Benjamin L Lindsley M Padhye A Geuser DM Ward TJ (2007) Phylogenetic diversity and micosphere array-based genotyping of human pathogenic fusaria, including isolates from the multistate contact lens - Associated US Keratitis outbreaks of 2005 and 2006. Journal of Clinical Microbiology 45: 2235–2248. 10.1128/JCM.00533-07 17507522 PMC1933018

[B29] Panicker S Ramraj B (2010) Studies on the epidemiology and control of Ascochyta blight of peas (*Pisum sativum* L) caused by *Ascochyta pinodes*. Archives of Phytopathology and Plant Protection 43: 51–58. 10.1080/03235400701652417

[B30] Quaedvlieg W Binder M Groenewald JZ Summerell BA Carnegie AJ Burgess TI Crous PW (2014) Introducing the consolidated species concept to resolve species in the *Teratosphaeriaceae*. Persoonia 33: 1–40. 10.3767/003158514X681981 25737591 PMC4312929

[B31] Ramaciotti Centre for Genomics (2019) Guide to Sanger Sequencing at RAMAC. https://www.ramaciotti.unsw.edu.au/sites/default/files/2019-04/RAMAC_Sanger_Sequencing_Service_Guide_2019_v1.0.pdf

[B32] Rayner RW (1970) A mycological colour chart. Commonwealth Mycological Institute, Kew.

[B33] Ronquist F Huelsenbeck JP (2003) MrBayes 3: Bayesian phylogenetic inference under mixed models. Bioinformatics 19: 1572–1574. 10.1093/bioinformatics/btg180 12912839

[B34] Salam MU Davidson JA Thomas GJ Ford R Jones RAC Lindbeck KD MacLeod WJ Kimber RBE Galloway J Mantri N (2011) Advances in winter pulse pathology research in Australia. Australasian Plant Pathology 40: 549–567. 10.1007/s13313-011-0085-3

[B35] Skoglund LG Harveson RM Chen W Dugan F Schwartz HF Markell SG Porter L Burrows ML Goswami R (2011) Ascochyta Blight of Peas. Plant Health Progress, 1–9. 10.1094/PHP-2011-0330-01-RS

[B36] Snyder WC Hansen HN (1947) Advantages of natural media and environments in the culture of fungi. Phytopathology 37: 420–421. 20249515

[B37] Soylu S Dervis S (2011) Determination of prevalence and incidence of fungal disease agents of pea (*Pisum sativum* L.) plants growing in Amik plain of Turkey. Research on Crops 12: 588–592.

[B38] Stamatakis A Alachiotis N (2010) Time and memory efficient likelihood-based tree searches on phylogenomic alignments with missing data. Bioinformatics 26: i132–i139. 10.1093/bioinformatics/btq205 PMC2881390 20529898

[B39] Sung GH Sung JM Hywel-Jones NL (2007) A multi-gene phylogeny of Clavicipitaceae (Ascomycota, Fungi): identification of localized incongruence using a combinational bootstrap approach. Molecular Phylogenetics and Evolution 44: 1204–1223. 10.1016/j.ympev.2007.03.011 17555990

[B40] Thambugala KM Daranagama DA Phillips AJL (2017) Microfungi on Tamarix. Fungal Diversity 82: 239–306. 10.1007/s13225-016-0371-z

[B41] Tran HS You MP Khan TN Barbetti MJ (2015) Pea black spot disease complex on field pea: dissecting the roles of the different pathogens in causing epicotyl and root disease. European Journal of Plant Pathology 144: 595–605. 10.1007/s10658-015-0798-1

[B42] Valenzuela-Lopez N Cano-Lira JF Guarro J Sutton DA Wiederhold N Crous PW Stchigel AM (2018) Coelomycetous *Dothideomycetes* with emphasis on the families *Cucurbitariaceae* and *Didymellaceae*. Studies in Mycology 90: 1–69. 10.1016/j.simyco.2017.11.003 29255336 PMC5725287

[B43] White TJ Bruns T Lee S (1990) Amplification and direct sequencing of fungal ribosomal RNA genes for phylogenetics. In: Innis MA Gelfand DH Sninsky JJe (Eds) PCR protocols: a guide to methods and applications.Academic Press, San Diego, USA, 315–322. 10.1016/B978-0-12-372180-8.50042-1

[B44] Wijayawardene NN Hyde KD Wanasinghe DN (2016) Taxonomy and phylogeny of dematiaceous coelomycetes. Fungal Diversity 77: 1–316. 10.1007/s13225-016-0360-2

[B45] Woudenberg JH De Gruyter J Crous PW Zwiers LH (2012) Analysis of the mating-type loci of co-occurring and phylogenetically related species of *Ascochyta* and *Phoma*. Molecular Plant Pathology 13: 350–362. 10.1111/j.1364-3703.2011.00751.x 22014305 PMC6638728

